# D, L-Sulforaphane Loaded Fe_3_O_4_@ Gold Core Shell Nanoparticles: A Potential Sulforaphane Delivery System

**DOI:** 10.1371/journal.pone.0151344

**Published:** 2016-03-16

**Authors:** Hamidreza Kheiri Manjili, Leila Ma’mani, Sharareh Tavaddod, Maedeh Mashhadikhan, Abbas Shafiee, Hossein Naderi-Manesh

**Affiliations:** 1 Department of Nanobiotechnology, Faculty of Biological Sciences, Tarbiat Modares University, Tehran, Iran; 2 Department of Nanotechnology, Agricultural Biotechnology Research Institute of Iran (ABRII), Agricultural Research, Education and Extension Organization (AREEO), Karaj, Iran; 3 Department of Biology, Faculty of Science, Science and Research Branch, Islamic Azad University, Tehran, Iran; 4 Department of Medicinal Chemistry, Faculty of Pharmacy and Pharmaceutical Sciences Research Center, Tehran University of Medical Sciences, Tehran 14176, Iran; Universidade Nova de Lisboa, PORTUGAL

## Abstract

A novel design of gold-coated iron oxide nanoparticles was fabricated as a potential delivery system to improve the efficiency and stability of d, l-sulforaphane as an anticancer drug. To this purpose, the surface of gold-coated iron oxide nanoparticles was modified for sulforaphane delivery via furnishing its surface with thiolated polyethylene glycol-folic acid and thiolated polyethylene glycol-FITC. The synthesized nanoparticles were characterized by different techniques such as FTIR, energy dispersive X-ray spectroscopy, UV-visible spectroscopy, scanning and transmission electron microscopy. The average diameters of the synthesized nanoparticles before and after sulforaphane loading were obtained ∼ 33 nm and ∼ 38 nm, respectively, when ∼ 2.8 mmol/g of sulforaphane was loaded. The result of cell viability assay which was confirmed by apoptosis assay on the human breast cancer cells (MCF-7 line) as a model of *in vitro*-cancerous cells, proved that the bare nanoparticles showed little inherent cytotoxicity, whereas the sulforaphane-loaded nanoparticles were cytotoxic. The expression rate of the anti-apoptotic genes (*bcl-2* and *bcl-x*_*L*_), and the pro-apoptotic genes (*bax* and *bak*) were quantified, and it was found that the expression rate of *bcl-2* and *bcl-x*_*L*_ genes significantly were decreased when MCF-7 cells were incubated by sulforaphane-loaded nanoparticles. The sulforaphane-loaded into the designed gold-coated iron oxide nanoparticles, acceptably induced apoptosis in MCF-7 cells.

## Introduction

Many studies confirmed that sulforaphane (SF) which was found in broccoli, cauliflower, kale and other cruciferous vegetables, and chemically was named 1-isothiocyanato-4-(methylsulfinyl)-butane, acts as an efficient incidence reducer in various types of tumors [1–3]. It has been revealed that SF has a promising and powerful anti-carcinogen effect in various cancers such as prostate, breast, osteosarcoma, bladder, pancreatic, hepatic, and melanoma [4–7]. SF as an antitumor agent acts via different mechanisms such as inhibiting angiogenesis and metastasis, inducing apoptosis, and reducing inflammation [8–11]. Despite the mentioned benefits of SF as an antitumor drug, its instability and sensitivity to oxygen, heat, and alkaline conditions, limits the usage of SF in pharmaceutical industries [12, 13].

In recent decades, nanoparticles (NPs) have opened bright horizons to develop new drug delivery systems (DDSs) due to their potential-biomedical-applications [14–17]. One type of nanoparticle is the magnetic NP which has various biomedical applications such as magnetic separator, magnetic-resonance-imaging contrast agent, and targeted drug deliver for cancer therapy [18–24]. The targeted drug delivery using magnetic NPs is a very useful technique to improve the performance of cancer treatment due to the several advantageous including the superparamagnetic property, inertness, ease of detection in the human body and high biocompatibility [25–27]. However, the naked iron oxide NPs are easily oxidized in air (especially magnetite). Therefore, in order to keep the stability of magnetic iron oxide NPs, it is important to develop protection strategies. These strategies comprise grafting or coating with polymers, biomolecules, organic molecules (small organic molecules or surfactants), or coating with an inorganic layer, such as silica, hydroxyapatite, metal, nonmetal elementary substance, metal oxide, or metal sulfide. Practically, it is worth to mention that in many cases the protecting shells not only stabilize the magnetic iron oxide NPs, but also will be used for further functionalization [28–30]. Regarding the mentioned points, Fe_3_O_4_@Au would be a good candidate as a drug delivery carrier.

By focusing on the medicinal properties of SF and in continuation of our experiences [31, 32], the aim of this study is to design and synthesis a nano-DDS based on [Fe_3_O_4_@Au] NPs to enhance the absorption and therapeutic level of SF as a promising antitumor drug. In order to pursue the drug delivery and absorption, FITC via thiolated polyethylene glycol was coated on the surface of gold-iron oxide core shell nanoparticle. Hence, we enabled to evaluate the efficiency of SF-delivery with fluorescence microscopy.

Since folate is essential for DNA synthesis and cell division and folic acid is an important vitamin required for healthy functioning of all cells, the folate in the cancer cells (divide more rapidly) is much more than the average amounts of folate in the normal cells. Therefore, folate receptors are often over expressed on the surface of many human cancerous cells including endometrial, renal, colon, and breast [33–35]. Thus, folic acid, that is a non-immunogenic ligand, has emerged as an attractive specific targeting molecule for anticancer drug delivery. Therefore, we propose a possible folic acid conjugated Fe_3_O_4_@Au NP by using a thiol molecule with the chemical formula (SH-PEG-NH_2_) as the linker to see whether the designed nano-DDS with folat could be a candidate for *in vitro* SF delivery or not. Herein, we evaluated *in vitro* SF-delivery by viability assay (MTT assay), fluorescence microscopy, and apoptosis assay (flow cytometry) of the human breast cancer cells (MCF-7 line) in the presence of free SF, FITC/FA@[Fe_3_O_4_@Au] NPs, SF-loaded FITC@[Fe_3_O_4_@Au] NPs, and SF-loaded FITC/FA@[Fe_3_O_4_@Au] NPs.

Our results indicate that the achieved nano-DDS appears as a promising candidate for *in vitro* study of SF delivery.

## Materials and Methods

### Cell line, culture conditions, reagents and instruments

All used chemicals were of analytical grade and purchased from commercial sources. 4’, 6’-diamidino-2-phenylindole (DAPI), FeCl_2_•4H_2_O, FeCl_3_•6H_2_O, HAuCl_4_•4H_2_O 99%, *d,l*-sulforaphane, 3-(4,5-dimethylthiazol-2-yl)-2,5-diphenyltetrazolium bromide (MTT), streptomycin, penicillin G, phosphate buffer (20 mM, *p*H = 7.8) and corresponding salts which were used throughout this research were obtained from Sigma-Aldrich or Merck. Fetal bovine serum (FBS) and Dulbecco’s Modified Eagle’s Medium (DMEM) as a culture medium and other supplements were obtained from Gibco (Germany).

MCF-7 cells were grown in DMEM medium supplemented with 10% (v/v) heat-inactivated FBS, 2% l-glutamine, 2.7% sodium bicarbonate, 1% Hepes buffer, and 1% penicillin-streptomycin solution (GPS, Sigma) at 37 ℃ in humidified atmosphere with 5% CO_2_. Cells were trypsinized in the solution of 0.05% trypsin and seeded into 96-well micro-plates at the density of 1 × 10^5^ cells/well. MCF-7 (human breast carcinoma) cell line was obtained from national cell bank of Iran (Pasteur institute, Iran) and cultured in DMEM medium containing 2 mM L-glutamine and 10% FBS, penicillin (50 IU/ml) and streptomycin (50 *μ*g/ml). Cells were separated using 0.1% trypsin and 10 *μ*M EDTA in phosphate-buffered solution.

The powder XRD spectrum was recorded at room temperature (RT) with a Philips X’pert 1710 diffractometer using Cu K*α* (*α* = 1.54056 A). The morphologies of the NPs were observed using SEM (Hitachi S-4800 II, Japan) equipped with energy dispersive X-ray spectroscopy (EDX). The TEM analysis was performed on a Hitachi H-7650 (Japan) operating at an acceleration voltage of 80 kv. The IR spectra were taken using Nicolet FT-IR Magna 550 spectrographs applying spectroscopic grade KBr. The size of NPs was assessed by DLS (Nano-ZS 90, Malvern Instrument, United Kingdom). The temperature was kept at 25 ℃ during the measuring process and measurements were recorded as the average of three test runs. The zeta potential of the NPs was measured in folded capillary cells using Nano sizer (Zeta sizer Nano ZS90, Malvern Instruments Ltd., Malvern, UK).

### Synthesis of NH_2_-PEG-SH

#### Synthesis of *α*-tosyl-*ω*-hydroxyl PEG (I)

To a solution of PEG (1450 g/mol, 3.45 mmol) in dry toluene, silver (I) oxide (5.2 mmol) and potassium iodide (0.7 mmol) were added, then p-toluene sulfonyl chloride (3.5 mmol) was added in one portion [36]. After 12 h stirring at RT, the mixture was filtered and the organic solvent evaporated. The product was dissolved in 10 ml dichloromethane (CH_2_Cl_2_) and then precipitated by drop-wise addition into diethyl ether (Et_2_O) and **I** was filtered. ^1^HNMR (DMSO): 7.79 (2H), 7.49 (2H), 4.56 (1H, OH), 4.11 (2H, CH_2_-OSO_2_), 3.49 (bs, PEG backbone), 2.43 (3H, s, CH_3_).

#### Synthesis of *α*-azide-*ω*-hydroxyl PEG (II)

A mixture of **I** (3.0 mmol) and NaN_3_ (4 mmol) in 150 ml of dry DMF was stirred overnight at 90 ℃ under argon atmosphere. The mixture was cooled down, filtered, and DMF was removed under vacuum. The product was dissolved in CH_2_Cl_2_ and washed twice with brine and water. The organic phase was dried over sodium sulfate and reduced to a small volume, and finally precipitated by dropping into Et_2_O. Then **II** was filtered. ^1^H-NMR (DMSO): 4.56 (1H, OH), 3.6 (2H, OCH_2_), 3.5 (bs, PEG backbone), 3.4 (2H, CH_2_-N_3_) [36].

#### Synthesis of *α*-azide-*ω*-thioacetate PEG (III)

To a mixture of **II** (3.0 mmol) in 100 ml CH_2_Cl_2_ were added NEt_3_ (10.0 mmol) and TsCl (10.0 mmol) and stirred overnight at RT. The solution was then filtered, and the filtrate was washed twice with saturated NH_4_Cl solution and water. Next, the organic phase was dried over sodium sulfate and filtered. The filtrate was concentrated by solvent evaporation, and then added drop-wise into dry Et_2_O and the product (*α*-azide-*ω*-tosyl PEG) was collected by filtration. Afterwards, freshly prepared sodium thioacetate (15.5 mmol) was added to a solution of *α*-azide-*ω*-tosyl PEG (3 mmol) in 100 ml dry DMF under inert atmosphere, and the mixture was stirred overnight at RT. After solvent evaporation, the residue was dissolved in CH_2_Cl_2_ and treated with active charcoal for 2 h. The mixture was filtered, and the filtrate was concentrated to small volume by rotary evaporation, and was added into dry Et_2_O. Then **III** was filtered. ^1^H-NMR (CDCl_3_): 3.63 (bs, PEG backbone), 3.58 (2H, OCH_2_), 3.38 (2H, CH_2_-N_3_), 3.07 (2H, CH_2_-S), 2.32 (3H, s, COCH_3_) [36].

### Synthesis of HS-PEG-NH_2_ (IV)

PPh_3_ (10.0 mmol) was added to a solution of **III** (3.0 mmol) in 50 ml dry MeOH, and the mixture was refluxed overnight under argon atmosphere. After cooling down the solvent removed by rotary evaporation, the resulting solid was dissolved in 10 ml CH_2_Cl_2_, and was added drop wise into dry Et_2_O and then **IV** was filtered. ^1^H-NMR (CDCl_3_): 3.88 (2H, CH_2_-CH_2_-S), 3.64 (bs, PEG backbone), 3.15 (2H, CH_2_-NH_2_), 2.67 (2H, CH_2_-SH), 1.56 (1H, SH) [36].

### Synthesis of HS-PEG-NH-FA (V)

Folic acid (0.1 mmol) was added to a mixture of dicyclohexyl carbodiimide (DCC) (0.15 mmol) and N-hydroxy succinimide (NHS) (0.12 mmol) in 3 ml of dry DMSO, the mixture was left to stir at RT overnight in the dark. After the removal of the byproduct, dicyclohexyl urea, the filtrate was mixed with **IV** (0.11 mmol) and stirred at RT for 24 h. Then **V** was precipitated by adding dry Et_2_O, dried in vacuum, and was stored at -20 ℃. ^1^HNMR (CD_3_OD): *δ* = 11.02 (1H, -COOH), 8.97 (1H, N = CH), 8.50-8.56 (m, 3H, 1NH_2_ and 1NH), 6.75-8.01 (m, 5H, 4CH and 1NH), 4.31-4.42 (m, 4H, 1CH_2_, 1CH, and 1NH), 3.78 (2H, CH_2_-CH_2_-SH), 3.58-3.64 (bs, PEG backbone), 3.20 (t, 2H, CH_2_-NH), 2.70 (t, 2H, CH_2_-SH), 2.05-2.10 (m, 4H, 2CH_2_), 1.56 (s, 1H, SH).

### Synthesis of HS-PEG-FITC (VI)

FITC (10.0 *μ*mol) and polymer **IV** (11.0 *μ*mol) was dissolved in 2 ml EtOH and 2 ml CHCl_3_ and stirred in the dark, under argon atmosphere. After 4 h, the product was precipitated by adding Et_2_O and dried. Then **VI** was filtered and stored at -20 ℃. ^1^HNMR (CD_3_OD): *δ* = 11.09 (s, 1H, 1COOH), 6.31-7.45 (m, 8H, 8CHAromatic), 6.18 (s, 1H, 1CHAromatic), 4.95 (bs, 1H, 1OH), 4.14 (bs, 1H, 1NH), 3.72 (t, 2H, CH_2_-CH_2_-SH), 3.56-3.64 (m, PEG backbone), 2.73 (t, 2H, CH_2_-SH), 1.56 (s, 1H, SH).

### Synthesis of Magnetic Nanocarrier

#### Synthesis of Fe_3_O_4_-Gold Core Shell Magnetic NPs

FeCl_2_•4H_2_O (2 ml, 2M) and FeCl_3_•6H_2_O (4 ml, 2M) were stirred at RT for 1 h, under argon atmosphere. Then, a concentrated solution of NaOH was drop-wise added under the inert atmosphere and the *p*H of the solution was carefully adjusted between 11 to 12. The solution was stirred at 20 ℃ for 1 h and then heated to 90 ℃ for another 1 h. Afterwards the system was kept at the same temperature for 1 h. Subsequently, Fe_3_O_4_ NPs were separated by using an external magnetic field and washed with of deionized water (DW) to achieve a neutral Fe_3_O_4_ suspension. Then the synthesis of [Fe_3_O_4_@Au] NPs was carried out according to Cui *et al.* [37] with some modifications. As-synthesized Fe_3_O_4_ NPs were applied as seed for the preparation of Fe_3_O_4_ gold core shell NPs. First, Fe_3_O_4_ NPs were dispersed in a micellar solution containing 0.1 M HAuCl_4_•4H_2_O solution, 3 g CTAB, 2.5 g 1-butanol and 15 g octane for 20 min using sonication, and then slowly mixed in a shaking incubator at 38 ℃ to allow the adsorption of Au^3+^ into the Fe_3_O_4_ surface. Then a solution of NaBH_4_ was added to the system as reducing agent and the mixture was incubated at RT in a shaking incubator. The formed NPs were washed with DW until neutralization. Finally, the resulting [Fe_3_O_4_@Au] NPs was filtered and dried under vacuum.

#### Synthesis of FITC/FA@[Fe_3_O_4_@Au] NPs

40 mg of [Fe_3_O_4_@Au] in 5 ml of dry CHCl_3_ was added to a suspension of 50 mg of **V** and 20 mg **VI** in CHCl_3_. The mixture was stirred 2 h in the dark under argon atmosphere at RT. The particles were then collected by applying an external magnetic device or centrifugation, and washed with CHCl_3_ and hexane (1:5 V/V). To obtain pure FITC/FA@[Fe_3_O_4_@Au] NPs, the product was dispersed in DW for dialysis for 24 h to remove unreacted organic molecules.

### Chemical and Physical Stability of FITC/FA@[Fe_3_O_4_@Au] NPs

The chemical stability of the functionalized [Fe_3_O_4_@Au] NPs under physiological conditions was carried out in DMEM media (*p*H = 7.4). FITC/FA@[Fe_3_O_4_@Au] NPs (5 mg/ml) was suspended in DMEM media at RT and placed inside the dialysis bag with a molecular cut-off of 14 kDa and then placed into DMEM cell culture media. The particles were stirred with sampling at 1, 2, 4, 6, 8, 12, 16 and 24 h. The samples were analyzed for FITC content using fluorescence spectrometer (perkin Elmer) at 488 nm. Increasing in FITC content outside the dialysis chamber indicates particle degradation. Therefore, at each time point 100 *μ*l of supernatant was picked up as a sample and then, 100 *μ*l of fresh medium was added to remain the volume at the previous scale. The absorbance of FITC was recorded, and the total release of FITC was calculated. To study physical stability of the synthesized NPs against aggregation, the particle sizes of FITC/FA@[Fe_3_O_4_@Au] NPs were assayed by DLS in PBS buffer. PBS represents the typical *p*H of physiological medium and is a very common biological buffer. The average hydrodynamic diameters of FITC/FA@[Fe_3_O_4_@Au] NPs was measured as a function of time up to 5 days.

### SF Loading and Releasing

FITC/FA@[Fe_3_O_4_@Au] NPs with the final concentration of 0.150 mg/ml was first sonicated with SF with an initial concentration of 0.300 mg/ml for 30 min and then stirred overnight at RT in the dark. The samples were separated applying an external magnet device, and washed with dry EtOH. The SF concentration in the liquid layer was measured using a standard SF concentration curve generated with an UV-visible spectrophotometer at 235 nm from a series of SF solutions with different concentrations. The drug loading efficiency (DLE) of FITC/FA@[Fe_3_O_4_@Au] NPs can be calculated by measuring differences between initial and residual SF concentrations as:

$$\begin{array}{c} \text{DLE}=\frac{{\text{W}}_{\text{Ini}}-{\text{W}}_{\text{Res}}}{{\text{W}}_{{}_{\text{FITC}/\text{FA}@[{\text{Fe}}_{3}O_{4}@\text{Au}]}}}, \end{array}$$ (1)

where *W*_Ini_, *W*_Res_, and *W*_FITC/FA@[Fe_3_O_4_@Au]_ are the weight of initial drug for loading, the weight of residual drug in solution after being loaded onto FITC/FA@[Fe_3_O_4_@Au] NPs, and the weight of FITC/FA@[Fe_3_O_4_@Au] NPs for loading, respectively. The release behavior of 5 mg of SF-loaded FITC/FA@[Fe_3_O_4_@Au] NPs in 5 ml of phosphate-buffered solution (PBS) at 37 ℃ was studied using a dialysis bag. At the time points of experiments, 0.5 ml of solution was collected and stored at 4 ℃ until detection. After each time of sampling, 0.5 ml of fresh PBS (*p*H = 7.4) was replaced to maintain the volume. Drug release was measured using the ultraviolet-visible spectrophotometer at 235 nm. Measuring of the release of SF from SF-loaded FITC/FA@[Fe_3_O_4_@Au] NPs was also repeated exactly in the case of citrate buffer (*p*H = 5.4). Meanwhile, the drug release were measured for the FITC/FA@[Fe_3_O_4_@Au] NPs as the control.

### Cell Viability by MTT Assay

To study the cytotoxicity of free SF, FITC/FA@[Fe_3_O_4_@Au] NPs, SF-loaded FITC@[Fe_3_O_4_@Au] NPs, and SF-loaded FITC/FA@[Fe_3_O_4_@Au] NPs in MCF-7 cell line; the colorimetric assay was applied to measure the reduction of MTT by mitochondrial succinate dehydrogenase. The MTT assay was performed at 24, 48, and 72 h of treatments. Cells were seeded into 96-well plates (10^4^ cells/well). After 24 h, diverse concentrations (range of 0.04-24 *μ*mol/l in DMEM) of free SF, FITC/FA@[Fe_3_O_4_@Au] NPs, SF-loaded FITC@[Fe_3_O_4_@Au] NPs, and SF-loaded FITC/FA@[Fe_3_O_4_@Au] NPs were applied. Then, after 24, 48, and 72 h of the treatment, the medium was removed and the cells were washed twice with PBS, and afterwards 20 *μ*l of MTT (5 mg/ml) was added to each well. Plates were incubated for 3 h at 37 ℃. Then, 100 *μ*l of DMSO was added to each well and left at 37 ℃ for 15 min. The optical density was measured at 570 nm using a micro-plate reader (Biotek, USA). The experiment of cytotoxicity of SF-loaded FITC/FA@[Fe_3_O_4_@Au] NPs in MCF-7 cell line after 24, 48, and 72 h of treatments were performed with six repeats, but the rest of the experiments with free SF, FITC/FA@[Fe_3_O_4_@Au], and SF-loaded FITC@[Fe_3_O_4_@Au] were performed in triplicate.

### Cellular Uptake by Fluorescence Microscopy

Cells were seeded into 6 well plates (10^5^ cells/well) and allowed to adhere. MCF-7 cells were considered as target. The cells were incubated at 37 ℃ at predetermined time points with free SF, FITC@[Fe_3_O_4_@Au] NPs, SF-loaded FITC@[Fe_3_O_4_@Au] NPs, and SF-loaded FITC/FA@[Fe_3_O_4_@Au] NPs with the final concentration of 3.5 *μ*mol/l. The cells were washed twice with PBS, before the digital-camera-microscopy. Observation was done under an inverted microscope (Olympus, IX81). The U-MWIB3 (BP-460-495 nm) filter was used to perform fluorescence microscopy. The digital images were captured with a DP72 digital camera. In order to indicate the intensity of FITC in the fluorescence images, images were analyzed using Matlab (The Math Works, Natick, MA). A colorbar was utilized to represent different intensity values.

### Cell Apoptosis by Flow Cytometry

Apoptosis of MCF-7 cells was studied by flow cytometry using Annexin-V/PI staining because phosphatidyl serine is unprotected and detectable by Annexin-V at the external surface of the cells at initial stages of apoptosis. Briefly, cells were seeded into 6 well plates (10^5^ cells/well) and were treated for 48 h with free SF, FITC/FA@[Fe_3_O_4_@Au] NPs, and SF-loaded FITC/FA@[Fe_3_O_4_@Au] NPs at IC_50_ concentration (24 *μ*mol/l). After incubation, the cells were washed twice with cold PBS and then re-suspended in 1× binding buffer (10 mM Hepes, 140 mM NaCl, and 2.5 mM CaCl_2_, *p*H = 7.2). 100 *μ*l of the solution transferred to 5 ml culture tube, and 5 *μ*l of Annexin V-FITC, and 5 *μ*l PI were added and incubated in dark situation for 15 min at RT. Then, 400 *μ*l of 1× binding buffer was added to each tube and analyzed by Flow Cytometry (Becton Dickinson FACS Canto II) within 1 h.

### Gene Expression Profile by Real-Time PCR

Total RNA of MCF-7 cells (10^5^ cells/ml) which were treated with 24 *μ*mol/l of free SF, FITC/FA@[Fe_3_O_4_@Au] NPs, SF-loaded FITC@[Fe_3_O_4_@Au] NPs, and SF-loaded FITC/FA@[Fe_3_O_4_@Au] NPs (72 h) was extracted by using TRIzol reagent according to the manufacturer’s instructions (Invitrogen Life Technologies). Total mRNA was directly processed to cDNA by reverse transcription with cDNA synthesis Kit (Fermentas) in a 25 *μ*l total volume reaction. The levels of mRNA of Bcl-2, Bcl-x_L_, Bak and Bax mRNA were quantified by the Real Time PCR while endogenous gene GAPDH were used as a control. Primers were designed using software primer express (Bcl-2 Forward: 5’-CAGGTTGAGGTTGTCCACAG-3’, Reverse: 5’-TGCTTATTCCAGAGCTCCCT-3’, Bcl-x_L_ Forward: 5’-AACTGTGGTTCAGTGTGGGA-3’, Reverse: 5’-CTAGCTTCCTCCAAGATGGC-3’, Bax Forward: 5’-GGGCAGTGTGATTTAGCAGA-3’, Reverse: 5’-TGAACCAAGATCATGCCATT-3’, Bak Forward: 5’-GGAAAGTTGCCCTGAGAAAG-3’, Reverse: 5’-TACTCAACACGCATGCAAGA-3’, GAPDH Forward: 5’-ATCATCCCTGCCTCTACTGG-3’, Reverse: 5’-GTCAGGTCCACCACTGACAC-3’). The thermal cycling conditions were as follows: initial denaturation at 95 ℃ for 10 min, followed by 45 cycles of 94 ℃ for 15 s, and 60 ℃ for 60 s. This program was followed by a melting curve program (60-98 ℃ with a heating rate of 0.3 ℃/s and continuous fluorescence measurement).

### Statistical Analysis

Matlab was used for all statistical analysis. Data are expressed as mean ± SEM. Statistical analysis to detect differences between groups was done by using one-way analysis of variance (ANOVA) with *p*-value <0.05 to indicate statistically significant differences. Post-hoc pairwise comparisons of Tukey(-Kramer) and Bonferroni tests were preformed and the smaller of the two intervals was taken.

## Results

### Preparation and Characterization of Nanocarrier

**Fig 1:**
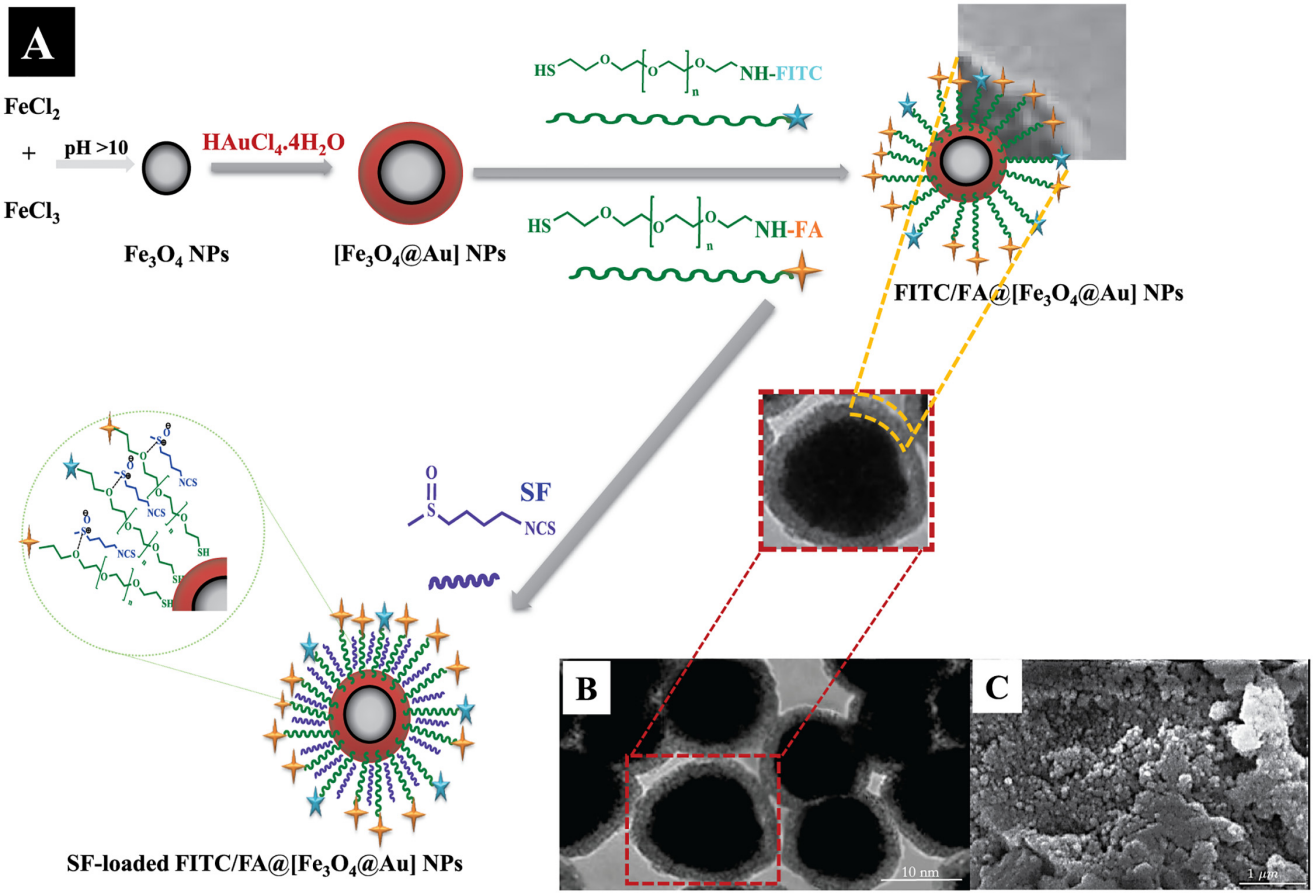
Schematic procedure of SF-loaded FITC/FA@[Fe_3_O_4_@Au] NPs synthesis (A), and TEM and SEM of NPs (B,C). A: Schematic procedure for synthesizing SF-loaded FITC/FA@[Fe_3_O_4_@Au] NPs. Abbreviations: PEG, poly(ethylene glycol); FA, folate; FITC, fluorescence isothiocyanate; SF, sulforaphane; NPs, nanoparticles. B: TEM of FITC/FA@[Fe_3_O_4_@Au] NPs. C: SEM of FITC/FA@[Fe_3_O_4_@Au] NPs.

The gold coated Fe_3_O_4_ NPs including [Fe_3_O_4_@Au], FITC/FA@[Fe_3_O_4_@Au] NPs, and SF-loaded FITC/FA@[Fe_3_O_4_@Au] NPs were prepared as shown in Fig 1A. [Fe_3_O_4_@Au] NPs were synthesized via co-precipitation of ferrous (Fe^2+^) and ferric (Fe^3+^) ions followed by coating resulting Fe_3_O_4_@ NPs with gold NPs. The surface of [Fe_3_O_4_@Au] NPs was decorated with thiolated polyethylene glycol-folic acid (HS-PEG-FA) and thiolated polyethylene glycol-Fluorescein isothiocyanate (HS-PEG-FITC) in order to fabricate FITC/FA@[Fe_3_O_4_@Au] NPs as an eligible magnetic nanocarrier. Due to the large affinity of thiol (S-H) function to the gold NPs, the thiolated PEG linkers are assembled around the [Fe_3_O_4_@Au] NPs through the self-assembly to achieve FITC/FA@[Fe_3_O_4_@Au] NPs.

TEM and SEM were applied to recognize the structural order and morphology of FITC/FA@[Fe_3_O_4_@Au] NPs (see Fig 1B and 1C). The analysis of SEM and TEM images showed that the average size of the synthesized NPs is less than 40 nm. The particle size distribution function confirmed that the size of NPs was 37±2.1 nm, which indicates a small heterogeneity in the size of the particles.

**Fig 2:**
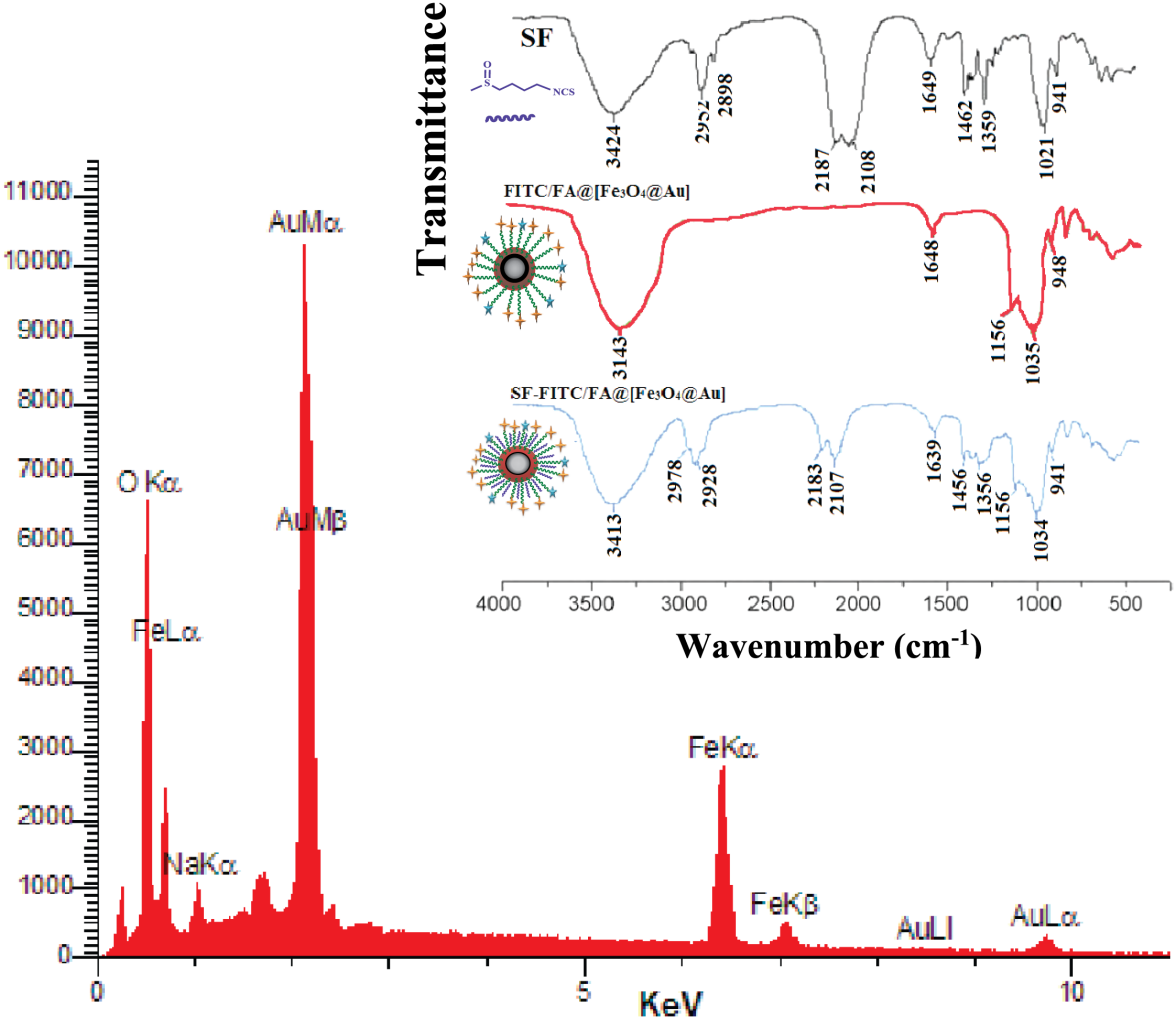
EDX and FTIR spectrum of NPs. The energy dispersive X-ray spectrum of [Fe_3_O_4_@Au] NPs. Inset: The FT-IR spectra of free SF, FITC/FA@[Fe_3_O_4_@Au] NPs, and SF-loaded FITC/FA@[Fe_3_O_4_@Au] NPs.

Energy dispersive X-ray (EDX) spectroscopy was done to investigate the presence of Au and Fe in the NPs. EDX spectrum of the NPs is presented in Fig 2, which confirms the existence of Au and Fe in the [Fe_3_O_4_@Au] NPs.

To confirm the existence of iron oxide-gold core shell, the powder X-Ray diffraction (XRD) experiment was used as an applicable technique. There were three bands in the XRD pattern which attributable to the corresponding reflections of the solid gold at 2*θ* = 44.67°, 51.72°, and 76.71° (JCPDS#89-3697). But, the characteristic bands of magnetic core no longer appeared in the XRD spectrum. The absence is due to the fact that all iron oxide NPs are coated with 2 nm thickness of gold [25, 38].

In order to confirm the successful loading of SF onto the FITC/FA@[Fe_3_O_4_@Au] NPs, Fourier transform infrared spectroscopy (FTIR) of free SF, FITC/FA@[Fe_3_O_4_@Au] NPs, and SF-loaded FITC/FA@[Fe_3_O_4_@Au] NPs was done (inset of Fig 2). In the spectrum of SF-loaded FITC/FA@[Fe_3_O_4_@Au] NPs, there are two strong absorbance picks at 2182 and 2109 cm^−1^. These absorbance picks are belonged to the stretching vibration of -N=C=S. Besides that, two other absorption peaks at 1452 and 1350 cm^−1^ are belonged to the deformation vibration of CH_3_.

The thermal gravimetric analysis (TGA) of FITC/FA@[Fe_3_O_4_@Au] NPs showed the first peak at 95 ℃ and the second peak at 395 ℃ which correspond to desorption of water and loss of the organic spacer group, respectively. Therefore, the loading of FITC/FA was about 20%. Following that, the TGA analysis of SF-loaded FITC/FA@[Fe_3_O_4_@Au] NPs showed the weight loss about 70%, which is attributed to removing SF and FITC/FA groups. Hence, in average 2.8 mmol/g of SF was loaded onto the surface of NPs.

**Fig 3:**
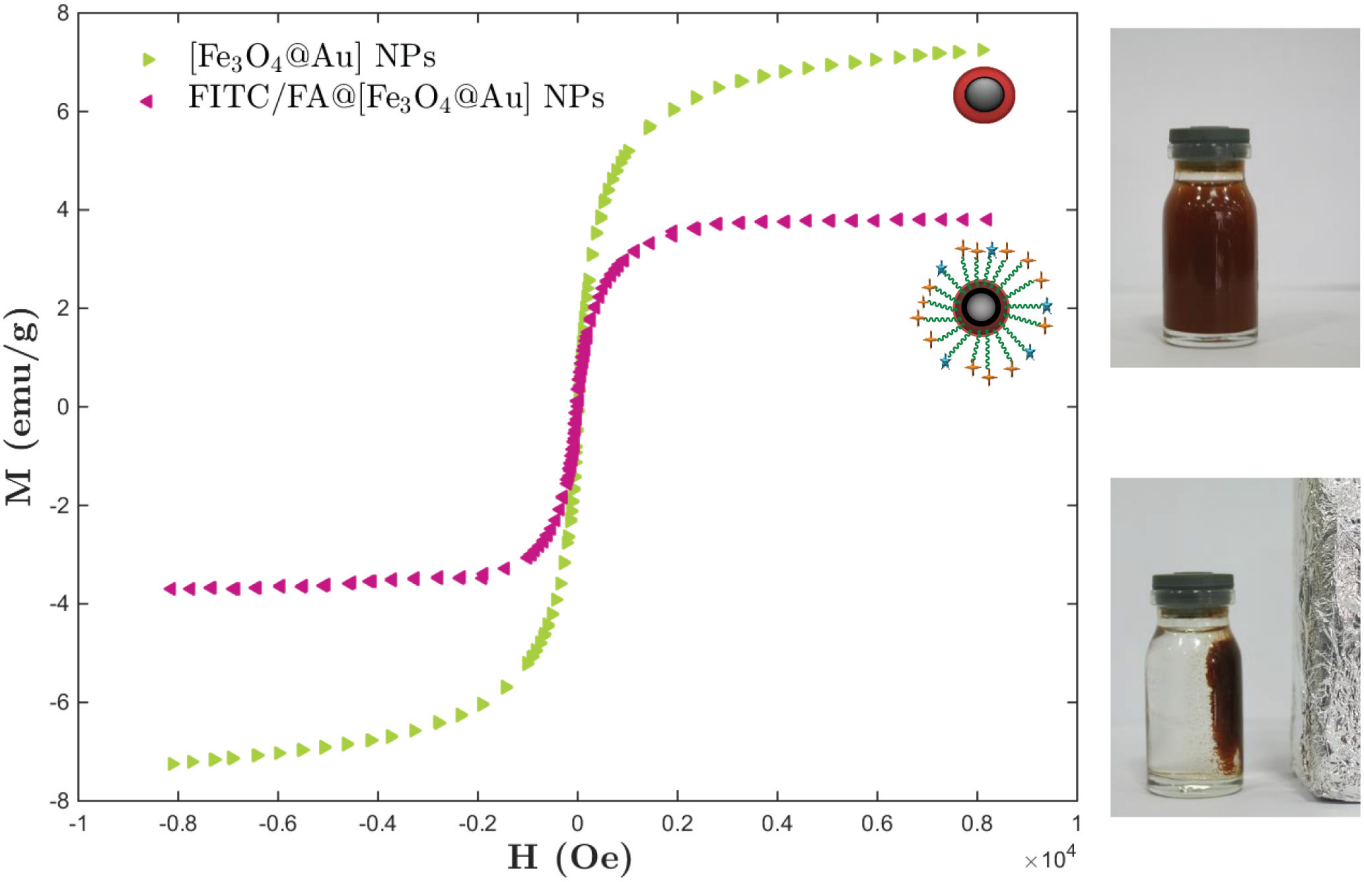
Magnetization hysteresis loop of NPs. The room-temperature magnetization (*M*) hysteresis loop curves of the same mass of [Fe_3_O_4_@Au] NPs and FITC/FA@[Fe_3_O_4_@Au] NPs as a function of applied magnetic field (*H*).

Since the existence of the magnetic NP in the designed NP would be a good candidate for *in vivo* therapy in animal model (using MRI), then it is important to synthesize the magnetic-core-shell with sufficient superparamagnetic property. To investigate it, the magnetic (*M*) hysteresis loop of the same mass of the [Fe_3_O_4_@Au] and FITC/FA@[Fe_3_O_4_@Au] NPs were investigated while magnetic field (*H*) at RT was applied. As shown in Fig 3, the value of magnetic saturation (*M*_*s*_) of the [Fe_3_O_4_@Au] and FITC/FA@[Fe_3_O_4_@Au] NPs were 7.24 and 2.07 emu/g at RT, respectively. The lower magnetic saturation of later NPs could be due to the influence of the PEG group. Furthermore, the hysteresis loop for the samples were completely reversible. The reversibility in hysteresis loop confirms that no aggregation imposes to the NPs in the external magnetic field and the NPs exhibit superparamagnetic property.

### Particle Size Distribution and Zeta Potential Measurement

The NPs with average diameter less than 100 nm are suitable to pass through cell membrane and works well for drug delivery. The mean diameter of the synthesized FITC/FA@[Fe_3_O_4_@Au] NPs and SF-loaded FITC/FA@[Fe_3_O_4_@Au] NPs were measured by dynamic light-scattering (DLS) technique. The mean size of the synthesized FITC/FA@[Fe_3_O_4_@Au] NPs and SF-loaded FITC/FA@[Fe_3_O_4_@Au] NPs were obtained 31.42±1.2 nm and 34.59±0.8 nm, respectively. The average size of both are less than 50 nm. Based on our measurement, NPs with and without drug are almost with small standard deviation in size (narrow particle size distribution function).

Another physical quantity which affects on cellular up take is the surface charge of synthesized NPs. The NPs with positive charge (size smaller than 100 nm) cross the cell membrane and readily taken up by cells [39]. The zeta potential of SF-loaded FITC/FA@[Fe_3_O_4_@Au] NP was measured and 9.47±4.6 mv was obtained which is suitable for cell membrane penetration.

### Chemical and Physical Stability of FITC/FA@[Fe_3_O_4_@Au] NPs

**Fig 4:**
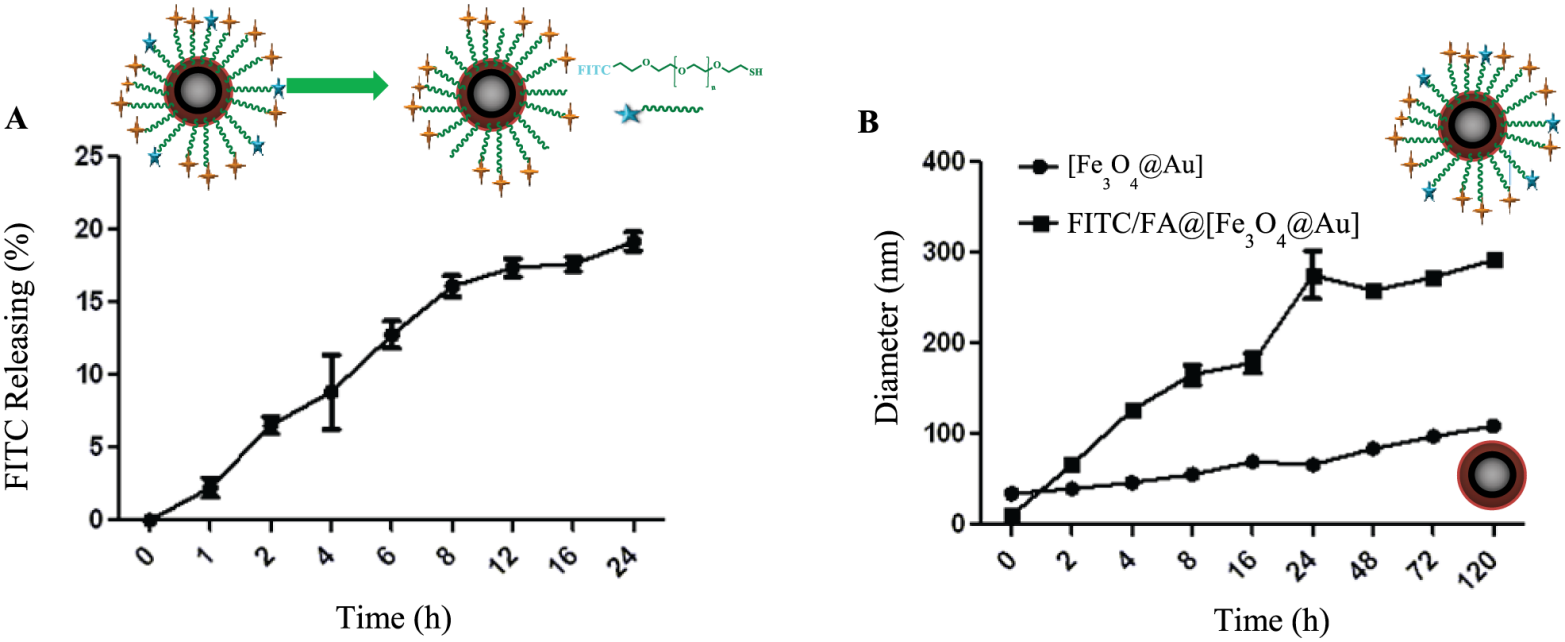
Release of FITC from NPs (A) and average hydrodynamic (B) of NPs. A: Release of FITC from FITC/FA@[Fe_3_O_4_@Au] NPs. B: The average hydrodynamic diameter of [Fe_3_O_4_@Au] and FITC/FA@[Fe_3_O_4_@Au] NPs at *p*H = 7.4.

To investigate the chemical stability of FITC/FA@[Fe_3_O_4_@Au] NPs, release of FITC from NPs was studied by UV-visible spectroscopy at 488 nm. The release of FITC could occurred due to the ligand exchange in the presence of a reductive media. The release profile was measured in two mediums including DMEM (Fig 4A) and glutathione (GSH). The releasing rate of FITC from FITC/FA@[Fe_3_O_4_@Au] during 24 h in DMEM shows a two-steps pattern. An initial burst release at the first 8 h which is followed by a relatively slower release during the next 16 h. At the first 8 h, the FITC-release was reached to around 17%, thereafter it remained almost steady through the next 16 h. At 24 h, the amount of FITC-release was about 18.87% in the cell culture medium (*p*H = 7.4). This implies that FITC/FA@[Fe_3_O_4_@Au] NPs which are suitable as drug carriers will reach to the cells with minimum lose of FITC (at 24 h).

The release of FITC moiety from the NPs in 5% GSH at 24 h showed no significant change in compare to DMEM. To the best of our knowledge, the disulfide bonds are cleaved by reducing agents such as GSH. Our synthesized NPs contains FITC moiety without any linkage S-S bond. Therefore, it is clear why there was not observed a significant change in the release profile of FITC from FITC/FA@[Fe_3_O_4_@Au] NPs.

To elaborate on the physical stability of FITC/FA@[Fe_3_O_4_@Au] NPs, the change in size of the NPs was studied. Change in size may occur during *in vitro* experiment. Therefore, the physical stability of the FITC/FA@[Fe_3_O_4_@Au] NPs at physiological *p*H was measured during 120 h. As the graph shows (see Fig 4B), the average diameter of FITC/FA@[Fe_3_O_4_@Au] NPs did not considerably change during 120 h in comparison with [Fe_3_O_4_@Au] NPs. Hence, from physical point of view the designed NPs were relatively stable.

### Drug Loading and Releasing

**Fig 5:**
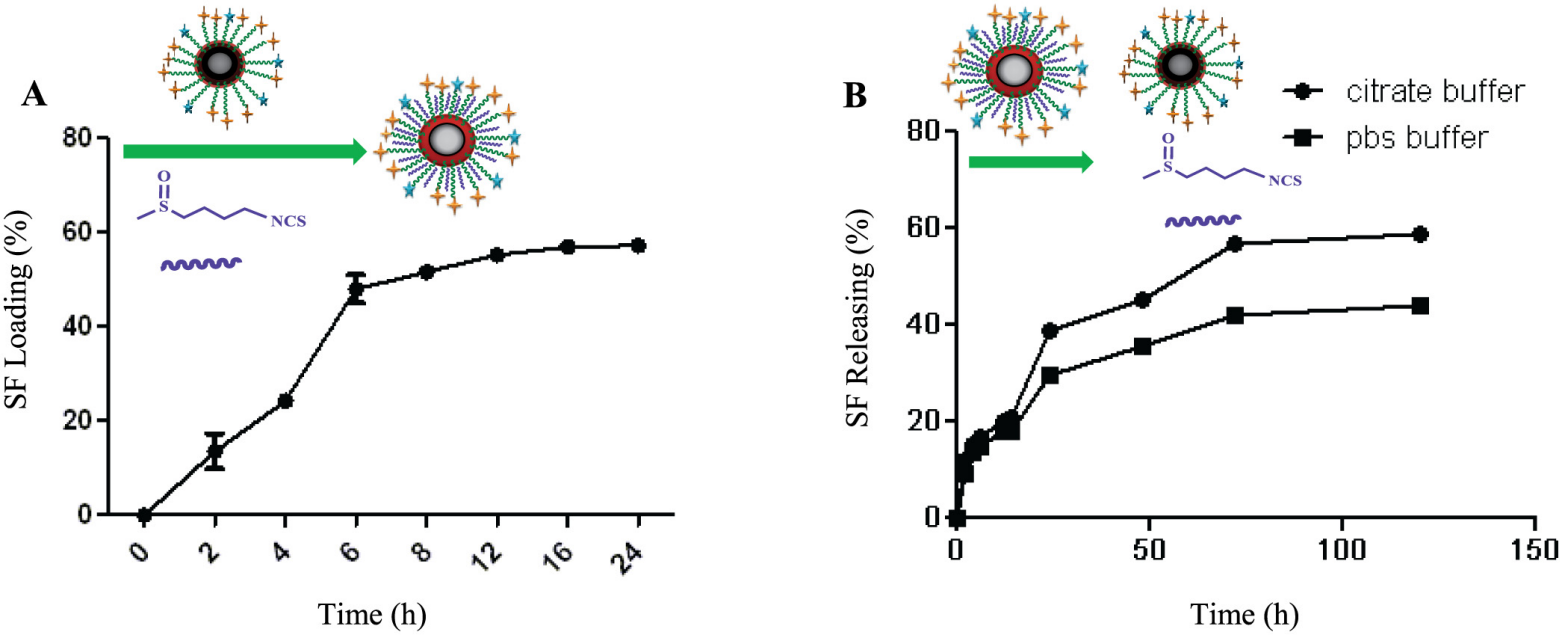
SF loading (A) and releasing (B). A: The profile of SF loading in FITC/FA@[Fe_3_O_4_@Au] NPs. B: The profile of SF releasing from SF-loaded FITC/FA@[Fe_3_O_4_@Au] NPs at two different *p*Hs (PBS buffer: *p*H = 7.4 and citrate buffer: *p*H = 5.4).

SF was confined in the organic PEG network around [Fe_3_O_4_@Au] NPs. As Fig 5A shows, the amount of SF-loaded onto the FITC/FA@[Fe_3_O_4_@Au] NPs could be reached up to 56% after 24 h.

Besides the SF loading, the release profile of SF from the surface of SF-loaded FITC/FA@[Fe_3_O_4_@Au] NPs was evaluated in two mediums including PBS and citrate buffer (Fig 5B). As it is shown in Fig 5B, at the same time (and longer than 72 h) of incubation SF-loaded FITC/FA@[Fe_3_O_4_@Au] NPs with MCF-7 cells, the release of SF form the NP at the *p*H of 5.4 (citrate buffer), about twenty percent is more than the release of SF form the NP at the *p*H of 7.4 (PBS).

### Cell Viability

**Fig 6:**
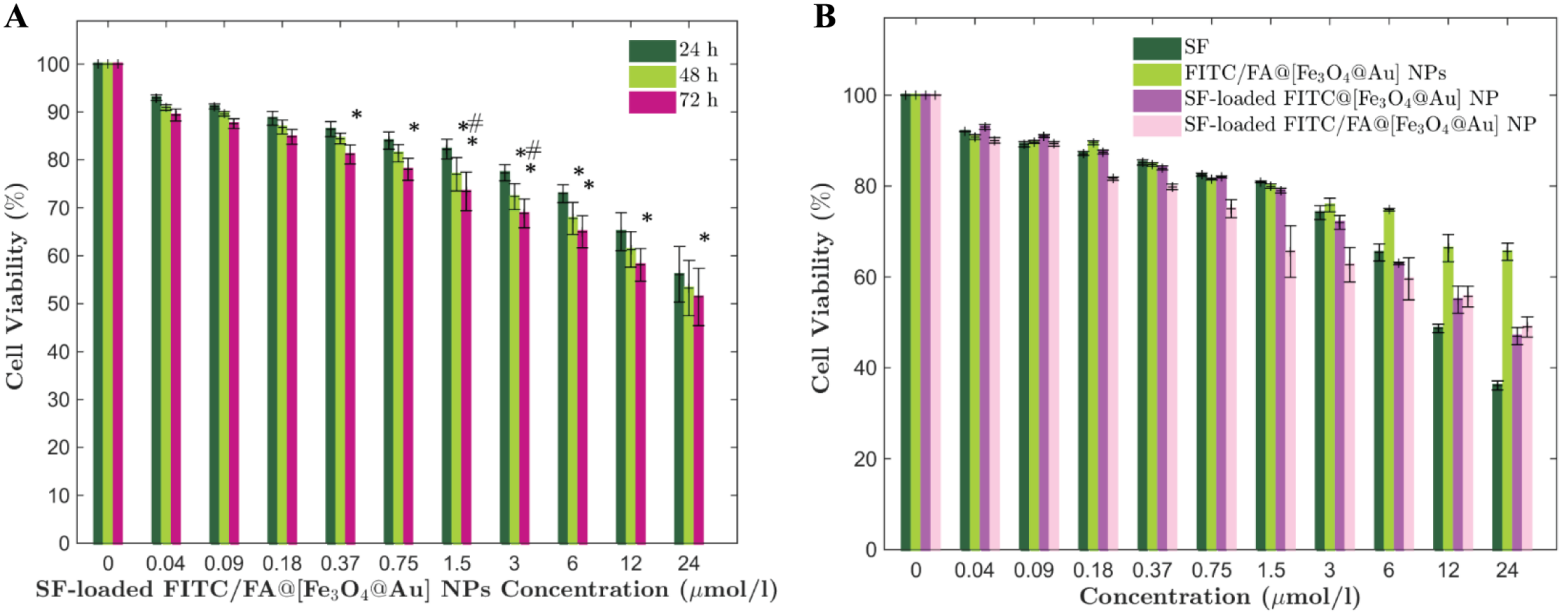
Viability of cells at different conditions. Viability of MCF-7 cells which were assessed A: after 24 h, 48 h, and 72 h of incubation with different concentrations of SF-loaded FITC/FA@[Fe_3_O_4_@Au] NPs. B: at different concentrations of incubation with free SF, FITC/FA@[Fe_3_O_4_@Au] NPs, SF-loaded FITC@[Fe_3_O_4_@Au] NPs, and SF-loaded FITC/FA@[Fe_3_O_4_@Au] NPs. Data are presented as the mean ± standard deviation of replicates.

To study the cytotoxicity of SF-loaded FITC/FA@[Fe_3_O_4_@Au] NPs, MCF-7 cells were incubated with different concentrations of SF-loaded FITC/FA@[Fe_3_O_4_@Au] NPs and the percentage of live cells after 24, 48, and 72 h of treatments were measured (Fig 6A). There is a significant (*p*-value <0.05) decrease in the percentage of cell viability at the concentrations of 1.5, 3 and 6 *μ*mol/l at 48 h versus 24 h. Also, there is a significant decrease (*p*-value <0.05) in the percentage of cell viability at the concentrations of 0.75, 1.5, 3, 6, 12 and 24 *μ*mol/l after 72 h versus 24 h. Finally, statistical analysis with the same *p*-value reveals that there is a significant decrease in the percentage of cell viability at the concentrations of 1.5 and 3 *μ*mol/l after 72 h versus 48 h. Furthermore, at any time, there is no significant decrease in the percentage of cell viability at concentrations less than 0.37 *μ*mol/l. Therefore, at concentrations lower than 1.5 *μ*mol/l, the effect of SF-loaded FITC/FA@[Fe_3_O_4_@Au] NPs is not considerable. Besides that, at the concentrations more than 6 *μ*mol/l of SF-loaded FITC/FA@[Fe_3_O_4_@Au] NPs, the cell viability did not change by time. Hence, it seems that the most effective concentration of SF-loaded FITC/FA@[Fe_3_O_4_@Au] NPs on cell viability is between 1.5 to 6 *μ*mol/l.

To verify the usefulness of the designed NPs as a potential drug carrier, at the concentration of SF-loaded FITC/FA@[Fe_3_O_4_@Au] NPs, which its efficiency on the cell viability was obtained before, the cytotoxicity of free SF, FITC/FA@[Fe_3_O_4_@Au] NPs, and SF-loaded FITC@[Fe_3_O_4_@Au] NPs were studied with the same procedure. As Fig 6B shows, at concentrations more than 12 *μ*mol/l, the SF can induce more cell death but inducing cell death with a high concentration of a drug (which is an unstable drug in our case) is not the aim of drug delivery system. The cytotoxicity of SF-loaded FITC/@[Fe_3_O_4_@Au] NPs significantly (*p*-value < 0.01) increases at the concentrations of 1.5, 3 and 6 *μ*mol/l versus FITC/FA@[Fe_3_O_4_@Au] NPs. Finally, the cytotoxicity of SF-loaded FITC/FA@[Fe_3_O_4_@Au] NPs becomes drastically significant (*p*-value <0.001) at concentrations of 1.5 and 3 *μ*mol/l. Both of the concentrations are in the rage of effective concentration of SF-loaded FITC/FA@[Fe_3_O_4_@Au] NPs which we obtained before (Fig 6A). To sum up, according to the cytotoxicity study using MTT assay, the FITC/FA@[Fe_3_O_4_@Au] NPs has a good effect as a vehicle for SF delivery at concentrations of 1.5 and 3 *μ*mol/l.

### Cellular Uptake

**Fig 7:**
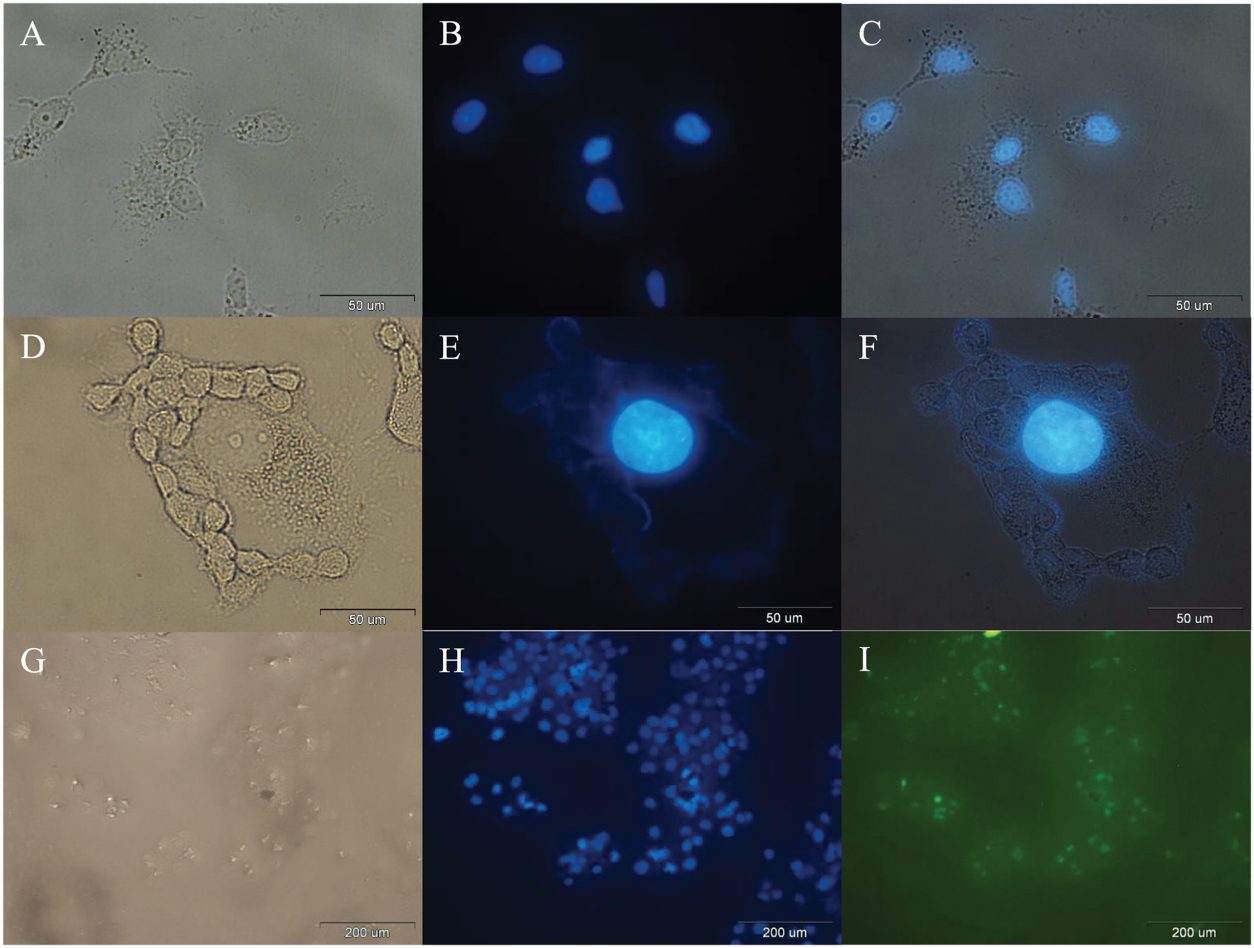
Optical microscopy images of cells after 24 h. A: Bright-field image, B: Fluorescence image of DAPI detection, and C: Combined of A and B, after 24 h incubation of MCF-7 cells (control). D: Bright-field image, E: Fluorescence image of DAPI detection, and F: Combined of D and E, after 24 h incubation of MCF-7 cells with free SF. G: bright-field image, H: fluorescence image of DAPI detection, and I: fluorescence image of FITC detection of the same area after 24 h incubation of MCF-7 cells with FITC@[Fe_3_O_4_@Au] NPs.

In order to investigate the cellular uptake, images of control cells (Fig 7A–7C), incubated cells with free SF (Fig 7D–7F), and incubated cells with FITC/FA@[Fe_3_O_4_@Au] NPs (Fig 7G–7I) were prepared. The emerged images of DAPI with bright field images (Fig 7B and 7E), and FITC-channel (Fig 7I) were prepared.

**Fig 8:**
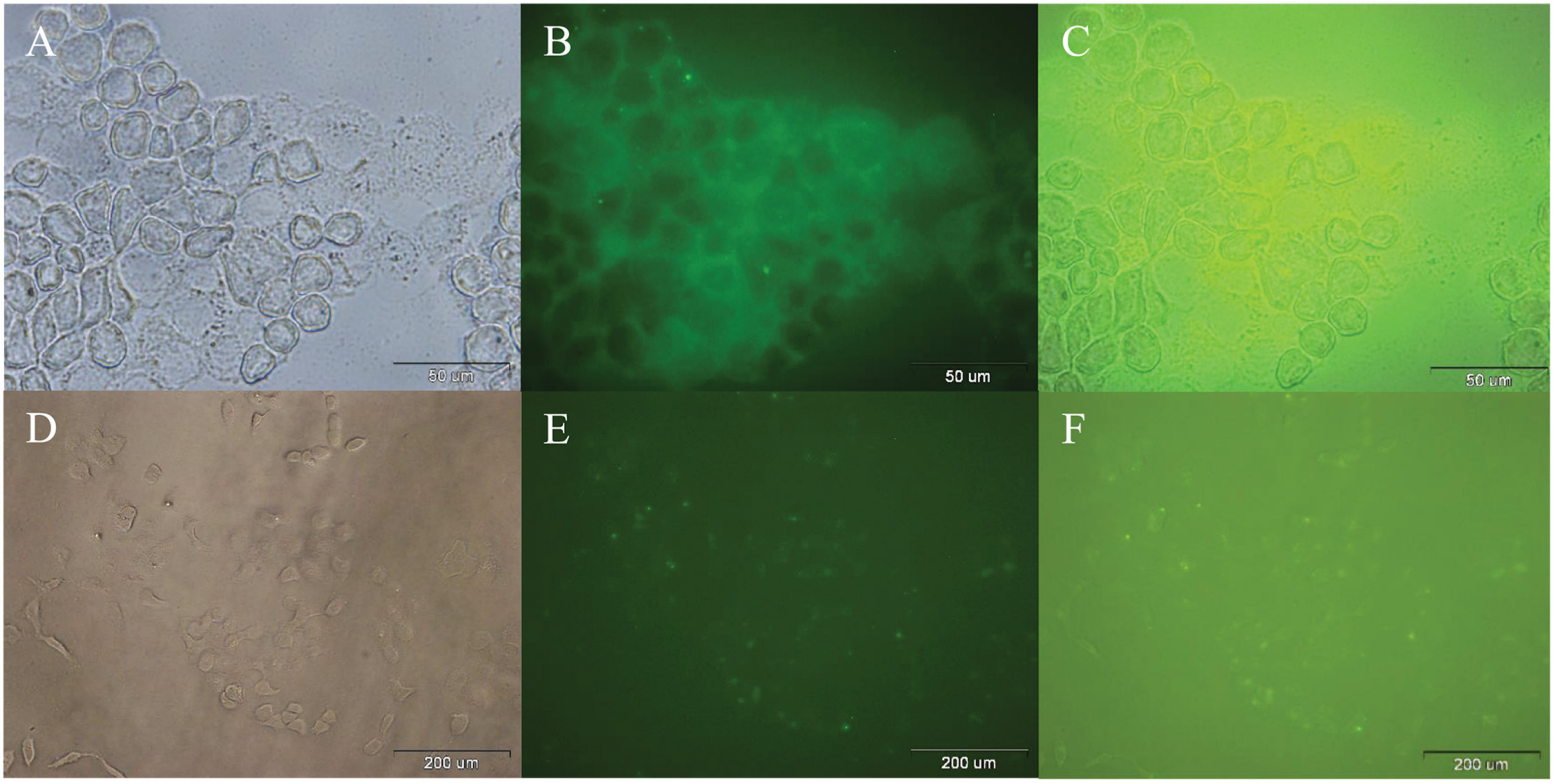
Optical microscopy images of incubated cells by SF-loaded FITC@[Fe_3_O_4_@Au] NPs and SF-loaded FITC/FA@[Fe_3_O_4_@Au] NPs after 24 h. A: Bright-field image, B: Fluorescence image of FITC detection, and C: Combined of A and B, after 24 h incubation of MCF-7 cells with SF-loaded FITC@/FA[Fe_3_O_4_@Au] NPs. D: Bright-field image, E: Fluorescence image of FITC detection, and F: Combined of D and E, after 24 h incubation of MCF-7 cells with SF-loaded FITC@[Fe_3_O_4_@Au] NPs.

Cellular uptake of SF-loaded FITC/FA@[Fe_3_O_4_@Au] NPs and SF-Loaded FITC@[Fe_3_O_4_@Au] NPs after 24 h incubation were investigated and detected by recording the bright-field image (Fig 8A and 8D), fluorescence image of FITC detection (Fig 8B and 8E), and emerged images of FITC with bright field images (Fig 8C and 8F), respectively. According to the Fig 8B and 8E, with the same concentration of SF-loaded FITC/FA@[Fe_3_O_4_@Au] NPs and SF-loaded FITC@[Fe_3_O_4_@Au] NPs, two properties of 1) homogenous (relatively) FITC-emission in the background, and 2) lower ratio (relatively) of the background-FITC-emission to the intercellular-FITC-emission indicate that the presence of the folate in FITC@[Fe_3_O_4_@Au] NPs, does not lead to a decrease in SF delivery and both proposed NPs deliver SF.

**Fig 9:**
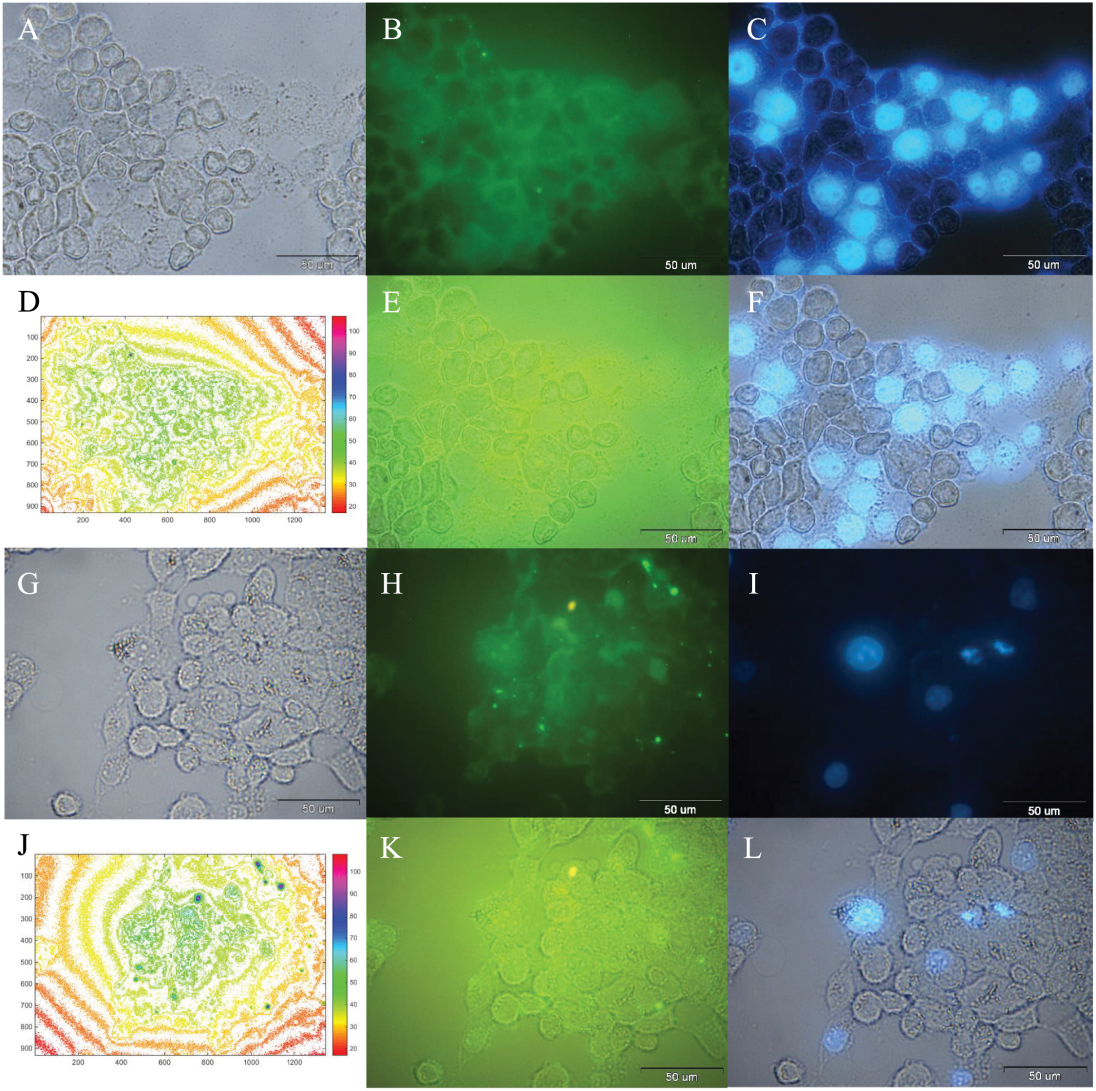
Optical microscopy images of incubated cells by SF-loaded FITC/FA@[Fe_3_O_4_@Au] NPs after 24 and 72 h. A: bright-field image, B: fluorescence image of FITC detection, and C: fluorescence image of DAPI detection of the same area after 24 h incubation of MCF-7 cells with SF-loaded FITC/FA@[Fe_3_O_4_@Au] NPs. D: The corresponding fluorescence intensity maps (colorbar shown) of FITC (in SF-loaded FITC/FA@[Fe_3_O_4_@Au] NPs) distribution of Fig 9B. E: Combined of A and B. F: Combined of A and C. G: bright-field image, H: fluorescence image of FITC detection, and I: fluorescence image of DAPI detection of the same area after 72 h incubation of MCF-7 cells with SF-loaded FITC/FA@[Fe_3_O_4_@Au] NPs. J: The corresponding fluorescence intensity maps (colorbar shown) of FITC (in SF-loaded FITC/FA@[Fe_3_O_4_@Au] NPs) distribution of Fig 9B. K: Combined of G and H. L: Combined of G and I.

The same method of microscopy was used for 72 h incubation of SF-loaded FITC/FA@[Fe_3_O_4_@Au] NPs with MCF-7 cells. As is shown in Fig 9A–9L, there is a one-to-one correspondence between live cells (DAPI staining) and nanoparticle/drug uptake by MCF-7 cells. Hence, the FITC/FA@[Fe_3_O_4_@Au] NPs were up taken by all (all in the field of view of the image) MCF-7 cells at 24 h of incubation (Fig 9A–9F). Based on the same colorbar corresponding the fluorescence intensity of FITC-emission, after 72 h, cells uptake more SF-loaded FITC/FA@[Fe_3_O_4_@Au] NPs. From the images it is possible to evaluate whether the drug release occurs intracellularly following NPs uptake or the drug release occurs extracellularly prior to NPs uptake/delivery. The dynamics of the drug release at two different *p*H were studied to estimate the percentage of releasing at different environmental conditions. Since the *p*H of the release medium is merely close to the *p*H of PBS at 24 h of incubation with SF-loaded FITC/FA@[Fe_3_O_4_@Au] NPs, about 30% of SF is released from SF-loaded FITC/FA@[Fe_3_O_4_@Au] NPs, extracellularly. It seems, probably during 24 h of incubation SF-loaded FITC/FA@[Fe_3_O_4_@Au] NPs with cells, the rest of the loaded drug (about 70%) enter via NPs, and release inside the cell.

### Cell Apoptosis

**Fig 10:**
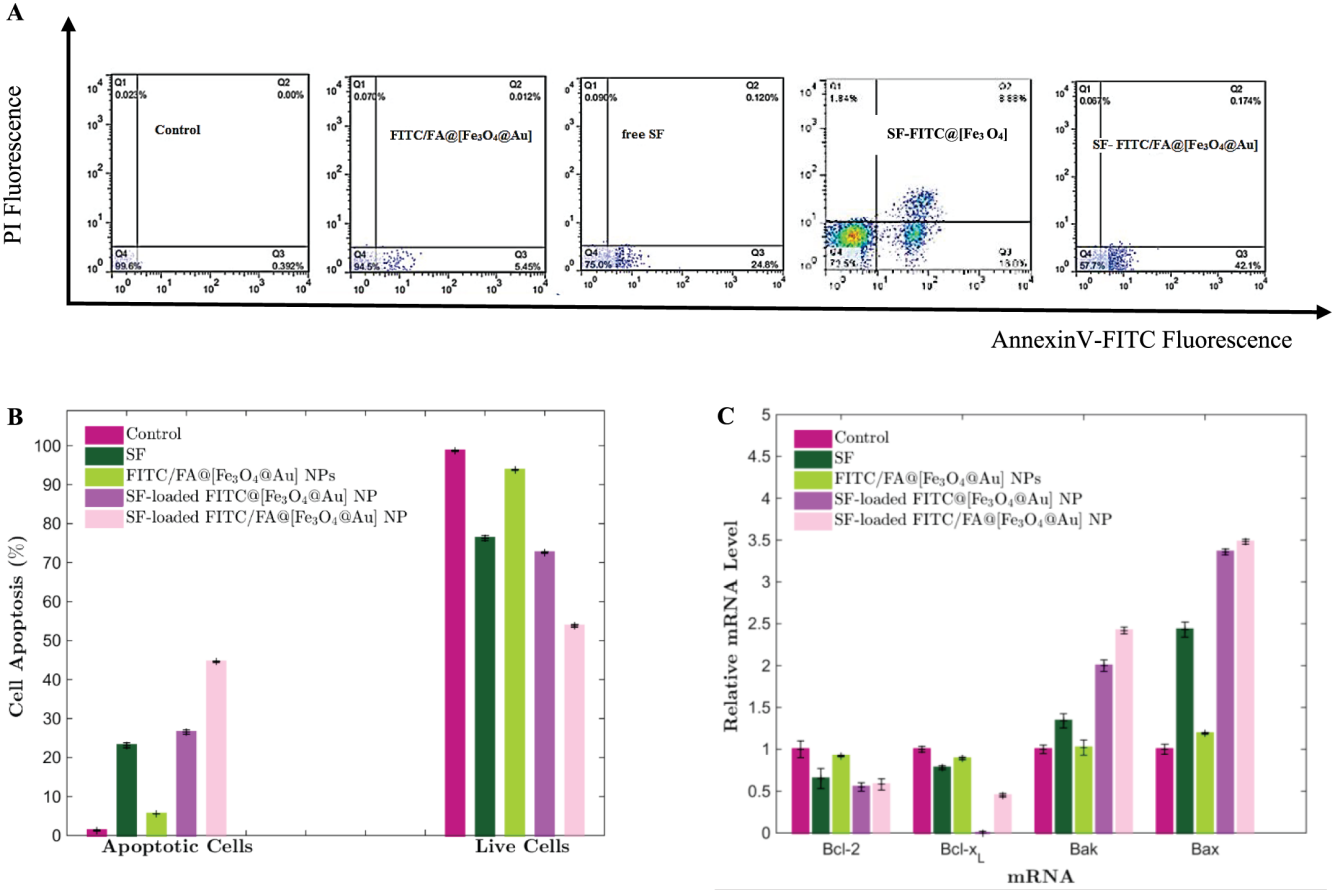
Flow cytometric analysis (A and B) and the rate of gene expression in MCF-7 cells (C). A: Flow cytometric analysis of MCF-7 cells were treated with free SF, FITC/FA@[Fe_3_O_4_@Au] NPs, SF-loaded FITC@[Fe_3_O_4_@Au] NPs, and SF-loaded FITC/FA@[Fe_3_O_4_@Au] NPs at equivalent SF concentration after 48 h. B: SF-loaded FITC/FA@[Fe_3_O_4_@Au] NPs induces significantly apoptosis versus free SF. Data are presented as the mean ± standard deviation of replicates. C: The rate of gene expression in MCF-7 cells after 72 h treatment with free SF, FITC/FA@[Fe_3_O_4_@Au] NPs, SF-loaded FITC@[Fe_3_O_4_@Au] NPs, and SF-loaded FITC/FA@[Fe_3_O_4_@Au] NPs.

A double staining flow cytometric assay using Annexin V/FITC (A), which reacts with viable cells, and propidium iodide (PI) was performed to quantify the apoptotic MCF-7 cells accurately. Fig 10A shows the percentages of MCF-7 cells viability (A^−^/PI^−^), early apoptosis (A^+^/PI^−^), late apoptosis (A^+^/PI^+^) and necrosis (A^−^/PI^+^) after treatment with free SF, FITC/FA@[Fe_3_O_4_@Au] NPs, SF-loaded FITC@[Fe_3_O_4_@Au] NPs and SF-loaded FITC/FA@[Fe_3_O_4_@Au] NPs. Statistic analysis of flow cytometric assay showed (Fig 10B) that the percentage of live cells (A^−^/PI^−^) were decreased significantly (P<0.0001) after treatment with free SF and SF-loaded FITC/FA@[Fe_3_O_4_@Au] NPs in compare to the untreated cells (control group), while the percentage of the treated cells with FITC/FA@[Fe_3_O_4_@Au] NPs, did not change significantly. Also, the percentages of total apoptosis (early + late apoptosis) in cells which were treated with free SF and SF- FITC/FA@[Fe_3_O_4_@Au] NPs increased significantly (P<0.0001) in comparison with control.

### Gene Expression Profile

Effects of SF-loaded FITC/FA@[Fe_3_O_4_@Au] NPs on the mRNA expression of selected genes was analyzed by Real-Time PCR. Statistical analysis of results (Fig 10C) indicate that after 72 h treatment with SF-loaded FITC/FA@[Fe_3_O_4_@Au] NPs, the expression rates of *bcl-2* and *bcl-x*_*L*_ (anti-apoptotic) genes significantly (P<0.05) were decreased while rates of mRNA expression of *bax* and *bak* (pro-apoptotic) (P<0.05) in comparison with control group.

## Discussion and Conclusions

We have designed, synthesized and characterized a novel and robust nano-magnetic gold DDS to improve the efficiency of SF delivery. The synthesized nano-magnetic vehicle was characterized by a number of techniques such as EDX, XRD, VSM, FT-IR, TGA, DLS, TEM, and SEM.

Owing to the hydrophilicity behavior of PEG function as surface modifier, the aforementioned surface manipulations provide the convenient surface to encompass SF via the electrostatic interaction between the S = O bond from SF and the oxygen groups from hydrophilic PEG linker. Hence, in order to 1) overcome the SF-instability, 2) improve SF-delivery, and 3) control SF-release, the surface of NPs was manipulated with functionalized PEG.

*In vitro* cytotoxicity study (MTT assay) of MCF- cells showed that SF-loaded FITC/FA@[Fe_3_O_4_@Au] NPs are more cytotoxic than free SF. The mechanism of induced appotosis in MCF-7 cells was revealed by double staining flow cytometric assay. Based on the statistical analysis of the obtained data, the rates of total apoptosis significantly were increased after loading onto the synthesized nano-magnetic vehicles in comparison with free SF. Following detection of apoptosis mechanism in cytotoxicity of SF-loaded FITC/FA@[Fe_3_O_4_@Au] NPs for MCF-7 cells, the expression rate of anti-apoptotic genes (*bcl-2* and *bcl-x*_*L*_) and pro-apoptotic genes (*bax* and *bak*) were quantified by Real-Time PCR technique. The expression rate of *bcl-2* and *bcl-x*_*L*_ genes significantly were decreased in comparison with untreated cells.

Notably, *in vitro* breast cancer study showed that FITC/FA@[Fe_3_O_4_@Au] NP would be a proper candidate nanocarrier for SF-delivery and provides a suitable and appropriate system for *p*H-dependent delivery of SF. These results would open new horizons to develop promising nano-DDSs to design further nanoparticle systems to improve the therapeutic effect of SF in the future.
